# Comparative analysis of three-year outcomes of different interbody fusion approaches for grade I-II lumbar spondylolisthesis with spinal stenosis

**DOI:** 10.3389/fsurg.2026.1847229

**Published:** 2026-10-12

**Authors:** Jian Luo, Li-Hua Shen, Teng-Fei Zhao, Chang-Shen Bao, Zhi-Chao Gao, Yong-Jun Meng, Guo-Fu Zheng

**Affiliations:** 1 Department of Orthopaedics, Linping Campus, Second Affiliated Hospital, Zhejiang University School of Medicine, Hangzhou City, China; 2 Department of Spinal Surgery, Jiefang Road Campus, Second Affiliated Hospital, Zhejiang University School of Medicine, Hangzhou City, China

**Keywords:** lumbar spinal stenosis, lumbar spondylolisthesis, oblique lumbar interbody fusion, transforaminal lumbar interbody fusion, unilateral biportal endoscopy

## Abstract

**Objective:**

This study aimed to compare surgical characteristics, complications, clinical and radiographic outcomes among patients undergoing unilateral biportal endoscopic posterior lumbar interbody fusion (UBE-PLIF), percutaneous endoscopic transforaminal lumbar interbody fusion (PE-TLIF), and oblique lumbar interbody fusion (OLIF) for grade I-II lumbar spondylolisthesis (LS) combined with lumbar spinal stenosis (LSS).

**Methods:**

From July 2019 to May 2022, patients with grade I-II LS combined with LSS were retrospectively included and grouped into the UBE-PLIF, PE-TLIF, and OLIF groups based on the surgical procedure. Baseline characteristics, surgical data, and complications were collected during hospitalization and follow-up. Clinical outcomes, including the Visual Analog Scale (VAS) for low back pain (VAS-back) and leg pain (VAS-leg), the Oswestry Disability Index (ODI), and the Japanese Orthopaedic Association (JOA) score, were assessed preoperatively and at 1, 6, 12, 24, and 36 months postoperatively. Radiographic outcomes, such as intervertebral disc height (IDH), slip percentage, lumbar lordosis angle, and fusion rate, were evaluated at the same timepoints.

**Results:**

The PE-TLIF and OLIF groups required longer operative times and greater blood loss than the UBE-PLIF group, with the OLIF group exhibiting the largest incision length, blood loss, and drainage volume (*P* < 0.05). After the 36-month follow-up, the UBE-PLIF group demonstrated significantly better VAS-leg scores at 24 and 36 months, as well as superior ODI and JOA scores at 36 months, compared to the other two groups (*P* < 0.05). Among radiographic outcomes, significant differences were found only in the interbody fusion rate at 6 months and IDH at 36 months (*P* < 0.05), with no differences in other parameters at the remaining timepoints. The total complication rates for UBE-PLIF, PE-TLIF, and OLIF were 13.53%, 18.94%, and 16.79%, respectively, with no significant intergroup differences (*P* > 0.05).

**Conclusion:**

UBE-PLIF, PE-TLIF, and OLIF demonstrate comparable safety and efficacy. UBE-PLIF is associated with less blood loss and modest advantages in selected late clinical outcomes, suggesting it is a viable alternative for grade I-II LS with LSS. Given the limitations of retrospective design, further validation through multicenter randomized controlled trials with longer follow-up is warranted.

## Introduction

Lumbar spondylolisthesis (LS) refers to the relative displacement of the upper and lower vertebrae caused by congenital or acquired factors, which mostly occurs at L4/5 or L5/S1, with a male to female ratio of approximately 2:1 (1). As one of the most prevalent spinal disorders in middle-aged and elderly populations, LS frequently coexists with lumbar spinal stenosis (LSS), disc herniation, and degenerative osteophytosis, manifesting as low back pain, hip pain, and lower extremity radicular symptoms (2). According to previous reports, the incidence of LS is about 6% in general population and up to 25% among elderly individuals, resulting in disability and a significant decline in quality of life (3–5). Over recent decades, evolving work patterns, lifestyle changes, and global population aging have contributed to a rising global incidence of LS, emerging as a notable public health concern (6–8). Building on prior work, 25%–37.9% of LS cases presented with concurrent LSS (9, 10), and this combination further exacerbates neurological damage symptoms such as pain, numbness, and intermittent claudication, increasing the difficulty of clinical treatment and the suffering in personal life.

In conventional spine surgery, open surgical decompression with lumbar interbody fusion is an established treatment for LS and LSS, effectively relieving neural compression, restoring spinal stability, and alleviating pain. Over the past two decades, with the development of endoscopic technology and minimally invasive techniques, minimally invasive surgery has been fully developed and has transformed surgical options, offering alternatives with decreased tissue damage, reduced blood loss, and accelerated recovery (11). Conventional lumbar decompression and fusion procedures such as posterior lumbar interbody fusion (PLIF), transforaminal lumbar interbody fusion (TLIF), and oblique lumbar interbody fusion (OLIF) are commonly used (12). Minimally invasive techniques have evolved from these conventional approaches, with each technique demonstrating its respective advantages and disadvantages. To our knowledge, there remains a paucity of comparative studies analyzing their mid- and long-term clinical outcomes in lumbar decompression and interbody fusion, which represents an area warranting further investigation. In clinical practice, unilateral biportal endoscopic posterior lumbar interbody fusion (UBE-PLIF), percutaneous endoscopic TLIF (PE-TLIF), and minimally invasive OLIF are three representative reduced-injury surgical techniques. Although numerous reports have shown that the three techniques are effective in treating LS, there is still insufficient evidence regarding patients with LS and concomitant LSS. This knowledge gap impedes evidence-based surgical decision-making. We hypothesized that while all three techniques are reliable surgical options for grade I-II LS with LSS, distinct performance profiles would be observed in their surgical characteristics, recovery trajectories, and follow-up outcomes. This study was therefore designed to directly compare the perioperative and follow-up clinical and radiographic outcomes of UBE-PLIF, PE-TLIF, and OLIF in patients with this condition.

## Patients and methods

### Patients

This retrospective study enrolled patients with grade I-II LS and concomitant LSS who underwent UBE-PLIF, PE-TLIF, or OLIF from a follow-up cohort between July 2019 and May 2022 in Hangzhou District. Diagnosis of LS and LSS followed current clinical guidelines (13, 14). Inclusion criteria were: (1) age ranging from 45 to 74 years after failure of ≥ 6 months of conservative treatment; (2) confirmed LS via flexion-extension radiographs, CT, or MRI, with instability defined as slip ≥ 4.5 mm or angular change ≥ 15°; and (3) LSS primarily caused by slippage, ligamentum flavum hypertrophy, or disc herniation. Exclusion criteria included: (1) previous lumbar surgery; (2) spondylolisthesis exceeding grade II; (3) severe osteoporosis (T-score ≤ −2.5 SD), ankylosing spondylitis, severe central osseous stenosis (sagittal diameter ≤ 10 mm), or other major spinal pathologies; (4) motor impairment from extra-spinal causes (e.g., stroke, severe hip/knee osteoarthritis, or poliomyelitis), cognitive impairment, malignant tumors, or other serious systemic diseases; and (5) missing key data, loss to follow-up, or study dropout. Sample size calculation was performed using G*Power based on a previous study (15). With the observed effect size of f = 0.282 (derived from ODI at 3-year follow-up), a two-sided significance level of 0.05, a power of 0.90, and three groups, the minimum sample size was estimated to be 165 patients (55 per group). The actual sample sizes (133, 132, 137 across groups) exceeded this threshold, indicating sufficient statistical power. After applying the inclusion and exclusion criteria, 153, 132, and 162 patients were identified in the UBE-PLIF, PE-TLIF, and OLIF groups, respectively. To ensure comparability of key confounding factors among the three groups and maximize sample retention, we used a restriction strategy based on the distribution of the UBE-PLIF cohort. Briefly, patients in the PE-TLIF and OLIF groups were included only if their age (±5 years), body mass index (±1.0 kg/m^2^), and lumbar bone mineral density T-score (±1) fell within the corresponding ranges of the UBE-PLIF group. Screening of eligible patients was independently performed by two researchers, and any disagreement was resolved by consultation with a third senior spinal surgeon. After applying the restriction strategy of the UBE-PLIF group distribution, 20 patients from the UBE-PLIF group and 25 from the OLIF group were excluded, whereas no patients were excluded from the PE-TLIF group. Consequently, 133, 132, and 137 patients in the three groups, respectively, were included in the final analysis.

### Surgical techniques and perioperative managements

All procedures were performed under general anesthesia by one of four experienced surgeons, with both the lead surgeon and the first assistant having performed over 100 prior cases to avoid learning curve effects. The UBE-PLIF and PE-TLIF groups were positioned prone, while the OLIF group was placed in the right lateral decubitus position. All implants, including PEEK or titanium cages, bone grafts, and pedicle screw systems, were supplied by Sanyou Medical Co., Ltd. (China) and CGBio Co., Ltd. (Korea), and were consistent across groups.

In the UBE-PLIF group, the procedure was performed according to Li et al. (16). Following prone positioning, two small bilateral incisions were created 2 cm from the midline, through which the working and viewing ports were established. Under continuous irrigation and endoscopic visualization, neural decompression was achieved by resecting the ligamentum flavum, partial lamina, and facet joint. After discectomy and endplate preparation, an interbody cage and bone graft were placed, followed by bilateral percutaneous pedicle screw fixation. The PE-TLIF procedure adhered to established techniques (17). A unilateral 1–2 cm incision was made on the symptomatic side. An endoscopic working channel was established, through which foraminoplasty, decompression, discectomy, and endplate preparation were performed. The cage and bone grafts were then implanted, followed by bilateral percutaneous screw fixation. For patients in the OLIF group, the technique described by Sardhara et al. (18) was followed. A 3-cm oblique incision was made anterior to the iliac crest. Blunt dissection provided retroperitoneal access, and a tubular retractor was positioned under electromyographic monitoring. Discectomy and endplate preparation were performed under direct visualization, followed by cage and bone graft implantation under fluoroscopy. Patients were then repositioned prone for bilateral percutaneous pedicle screw fixation.

Postoperatively, all patients received standard prophylactic antibiotics and symptomatic treatment, including analgesia, anti-inflammatory therapy, and neurotrophic agents. The drainage tube was removed once output fell below 30 mL per day. During bed rest, patients performed axial turning and lower-limb exercises under guidance, with early ambulation using a lumbar brace encouraged.

### Study variables

Baseline characteristics (including age, gender, body mass index, as detailed in Table 1) and surgical characteristics (including operation time, total incision length, cage type, as detailed in Table 2) were documented. Intraoperative blood loss was quantified as (suction volume – irrigation volume) + gauze weight gain (1 g = 1 mL) (19). Clinical outcomes were assessed using the Visual Analog Scale for back (VAS-back, 0–10 points) and leg pain (VAS-leg, 0–10 points), the Oswestry Disability Index (ODI, 0%–100%), and the Japanese Orthopaedic Association score (JOA, 0–29 points), evaluated preoperatively and at 1, 6, 12, 24, and 36 months postoperatively according to previous protocols (20). Radiographic outcomes were evaluated at predefined intervals using established methods, which included the intervertebral disc height (IDH, measured by Dabbs’ method), slip percentage (calculated as the ratio of vertebral slip), lumbar lordosis angle (Cobb angle between the upper endplates of L1 and S1), and fusion success rate (Bridwell grades I–II; indeterminate cases resolved by CT) (21). Complications were categorized as major or minor. Major complications encompassed events such as permanent neurological deficit, reoperation, or other life-threatening conditions. Minor complications included transient or mild events that did not lead to significant sequelae (22). For ambiguous cases, the Clavien-Dindo classification was incorporated, designating grades ≤ II as minor and grades > II as major (23). Each patient was evaluated independently by two surgeons, with a third senior surgeon resolving substantial disagreements. To assess reliability, 50 patients were randomly re-evaluated after a cooling-off period (> 3 months). The analysis showed reliable agreement: intra- and inter-observer intraclass correlation coefficients for continuous variables (JOA, IDH, slip percentage, and lumbar lordosis angle) were 0.87–0.95 and 0.76–0.90, respectively, while weighted Kappa values for interbody fusion rate were 0.92 and 0.89. For two-level procedures, IDH and slip values were averaged across segments.

**Table 1:** Comparison of baseline characteristics among the three groups [($\bar{x}$ ± s), n(%)].

Baseline characteristics	UBE-PLIF (*n* = 133)	PE-TLIF (*n* = 132)	OLIF (*n* = 137)	*F/χ^2^/H*	*P*
Age (years)	59.34 ± 11.31	61.15 ± 9.87	58.77 ± 10.34	1.863	0.157
Gender					
Male	57 (42.86)	60 (45.45)	52 (37.96)	1.606	0.448
Female	76 (57.14)	72 (54.55)	85 (62.04)		
Body mass index (kg/m^2^)	25.85 ± 4.15	26.02 ± 3.89	24.98 ± 4.03	2.599	0.076
Duration of lumbar disease (years)					
< 1	38 (28.57)	44 (33.33)	41 (29.93)	3.265	0.515
1–3	53 (39.85)	42 (32.82)	43 (31.39)		
> 3	42 (31.58)	46 (34.85)	53 (38.69)		
Affected segments					
L3/L4	13 (9.77)	12 (9.09)	16 (11.68)	3.772	0.708
L4/L5	80 (60.15)	69 (52.27)	73 (53.28)		
L5/S1	35 (26.32)	42 (31.82)	38 (27.74)		
L3/L4 + L4/L5 or L4/L5 + L5/S1	5 (3.76)	9 (6.82)	10 (7.30)		
Etiological type					
Degenerative spondylolisthesis	97 (72.93)	91 (68.94)	89 (64.96)	2.000	0.368
Isthmic spondylolisthesis	36 (27.07)	41 (31.06)	48 (35.04)		
Meyerding grade					
Ⅰ	91 (68.42)	87 (65.91)	86 (62.77)	0.960	0.619
Ⅱ	42 (31.58)	45 (34.09)	51 (37.23)		
Comorbidities					
Hypertension	38 (28.57)	47 (35.61)	50 (36.50)	2.261	0.323
Diabetes	24 (18.05)	29 (21.97)	26 (18.98)	0.706	0.703
Heart disease	22 (16.54)	26 (19.70)	30 (21.90)	1.249	0.536
Other chronic diseases	15 (11.28)	22 (16.67)	17 (12.41)	1.842	0.398

UBE-PLIF, unilateral biportal endoscopic posterior lumbar interbody fusion; PE-TLIF, percutaneous endoscopic transforaminal lumbar interbody fusion; OLIF, oblique lumbar interbody fusion.

**Table 2:** Comparison of surgical characteristics among the three groups [($\bar{x}$ ± s), n(%)].

Surgical characteristics	UBE-PLIF (*n* = 133)	PE-TLIF (*n* = 132)	OLIF (*n* = 137)	*F/χ^2^/H*	*P*
Operation time (min)	160.69 ± 38.83	178.09 ± 43.74^a^	181.46 ± 40.12^b^	9.933	< 0.001
Total incision length (cm)	6.89 ± 1.24	6.90 ± 1.10	8.71 ± 1.60^b^^,^^c^	83.641	< 0.001
Cage type					
Titanium alloy	65 (48.87)	70 (53.03)	81 (59.12)	2.892	0.236
Polyether-ether-ketone	68 (51.13)	62 (46.97)	56 (40.88)		
Proportion of allogeneic bone					
< 30%	24 (18.05)	22 (16.67)	30 (21.90)	1.812	0.770
30%–60%	73 (54.89)	75 (58.58)	69 (50.36)		
> 60%	36 (27.07)	34 (25.76)	38 (27.74)		
Estimated blood loss (mL)	123.62 ± 37.85	158.62 ± 42.14^a^	182.39 ± 46.52^b^^,^^c^	65.604	< 0.001
Drainage volume (mL)	97.29 ± 31.47	102.67 ± 35.75	140.80 ± 41.20^b^^,^^c^	57.502	< 0.001
Time to ambulation (d)	2.87 ± 0.90	2.94 ± 1.09	3.13 ± 1.26	2.044	0.131
Lead surgeon + First assistant					
ZG + JL	41 (30.83)	38 (28.79)	40 (29.20)	3.710	0.882
TZ + JL	36 (27.07)	29 (21.97)	35 (25.55)		
GZ + BC	20 (15.04)	25 (18.94)	19 (13.87)		
YM + GZ	19 (14.29)	17 (12.88)	23 (16.79)		
Other combinations	17 (12.78)	23 (17.42)	20 (14.60)		
Hospital stay (d)	10.14 ± 2.86	9.88 ± 3.01	10.39 ± 3.17	0.960	0.384
Hospitalization costs (USD)	8,274.31 ± 1,789.18	8,094.76 ± 2,035.92	7,863.25 ± 1,978.40	1.529	0.218

UBE-PLIF, unilateral biportal endoscopic posterior lumbar interbody fusion; PE-TLIF, percutaneous endoscopic transforaminal lumbar interbody fusion; OLIF, oblique lumbar interbody fusion.

a PE-TLIF vs. UBE-PLIF, *P* < 0.05.

b OLIF vs. UBE-PLIF, *P* < 0.05.

c OLIF vs. PE-PLIF, *P* < 0.05.

#### Statistical analysis

Statistical analysis of data was performed using SPSS 26.0 software. Normality of continuous variables was assessed using Shapiro–Wilk test. The continuous data in line with normal distribution were presented as mean ± standard deviation (x̄ ± s), which were analyzed by analysis of variance among the three groups, followed by the *SNK-q* test for pairwise comparisons. The categorical data were presented as frequencies and percentages (%) and were analyzed by the chi-square (*χ^2^*) test or Fisher's exact test. The ordinal data were analyzed by the Kruskal–Wallis H test. A *P*-value < 0.05 was considered statistically significant.

## Results

### Comparison of baseline characteristics

There were no significant differences in baseline characteristics, including age, gender, body mass index, duration of lumbar disease, affected segments, etiological type, Meyerding grade, or comorbidities among the three groups (*P* > 0.05), as shown in Table 1.

### Comparison of surgical characteristics

No significant differences were observed among the three groups in cage type, proportion of allogeneic bone, time to ambulation, primary surgical operators (lead surgeon and first assistant), hospital stay, or costs (*P* > 0.05). However, operative time was longer in the PE-TLIF and OLIF groups than in the UBE-PLIF group, and total incision length was greater in the OLIF group compared to the other two groups (*P* < 0.05). The OLIF group also showed the highest estimated blood loss and drainage volume, while the PE-TLIF group had greater blood loss than the UBE-PLIF group (*P* < 0.05), as shown in Table 2.

### Comparison of clinical outcomes

No significant differences were found in preoperative VAS-back, VAS-leg, ODI, or JOA scores among the three groups (*P* > 0.05). Throughout the three-year follow-up, VAS-back scores showed no intergroup differences at any timepoint (*P* > 0.05). Similarly, VAS-leg, ODI, and JOA scores at 1–12 months did not differ significantly (*P* > 0.05). However, the UBE-PLIF group demonstrated significantly lower VAS-leg scores at 24 and 36 months compared to both the PE-TLIF and OLIF groups (*P* < 0.05). Moreover, significantly lower ODI and higher JOA scores were observed in the UBE-PLIF group compared to the PE-TLIF group at 24 and 36 months, as well as against the OLIF group at 36 months (*P* < 0.05), as shown in Table 3 and Figure 1.

**Table 3:** Comparison of clinical outcomes among the three groups ($\bar{x}$ ± s).

Clinical outcomes		UBE-PLIF (*n* = 133)	PE-TLIF (*n* = 132)	OLIF (*n* = 137)	*F*	*P*
VAS-back (points)						
Preoperatively	Admission	7.09 ± 1.53	6.89 ± 1.61	6.94 ± 1.43	0.620	0.539
Postoperatively	1 month	2.80 ± 1.19	3.04 ± 1.42	3.11 ± 1.25	2.132	0.120
	6 months	1.86 ± 0.79	2.08 ± 0.90	1.95 ± 0.81	2.328	0.099
	12 months	1.58 ± 0.85	1.76 ± 0.77	1.64 ± 0.90	1.568	0.210
	24 months	1.46 ± 0.69	1.58 ± 0.73	1.52 ± 0.65	1.001	0.368
Final follow-up	36 months	1.33 ± 0.72	1.25 ± 0.64	1.17 ± 0.56	2.092	0.125
VAS-leg (points)						
Preoperatively	Admission	5.91 ± 1.54	6.12 ± 1.70	5.73 ± 1.97	1.675	0.189
Postoperatively	1 month	2.86 ± 1.15	3.02 ± 1.23	3.17 ± 1.09	2.422	0.090
	6 months	1.70 ± 0.86	1.79 ± 0.96	1.80 ± 0.84	0.515	0.598
	12 months	1.49 ± 0.64	1.60 ± 0.71	1.62 ± 0.73	1.360	0.258
	24 months	1.28 ± 0.57	1.58 ± 0.87^a^	1.51 ± 0.76^b^	5.922	0.003
Final follow-up	36 months	0.96 ± 0.51	1.42 ± 0.72^a^	1.30 ± 0.65^b^	18.898	< 0.001
ODI (%)						
Preoperatively	Admission	58.25 ± 11.63	56.82 ± 9.12	55.94 ± 11.15	1.599	0.203
Postoperatively	1 month	28.53 ± 7.16	29.67 ± 6.64	30.20 ± 7.38	1.958	0.142
	6 months	19.84 ± 5.58	20.90 ± 7.41	21.13 ± 6.28	1.519	0.220
	12 months	15.58 ± 5.76	16.34 ± 6.45	17.06 ± 5.07	2.213	0.111
	24 months	11.86 ± 4.54	13.23 ± 5.26^a^	12.72 ± 4.60	2.751	0.065
Final follow-up	36 months	9.61 ± 3.89	12.65 ± 4.83^a^	11.63 ± 4.51^b^	16.209	< 0.001
JOA score (points)						
Preoperatively	Admission	12.71 ± 4.10	13.43 ± 3.96	12.45 ± 5.01	1.788	0.169
Postoperatively	1 month	17.08 ± 3.67	16.94 ± 4.09	17.55 ± 4.28	0.853	0.427
	6 months	24.37 ± 2.74	23.86 ± 3.23	24.41 ± 3.08	1.369	0.255
	12 months	24.89 ± 2.52	24.57 ± 2.47	25.10 ± 2.12	1.695	0.185
	24 months	25.08 ± 1.95	24.19 ± 2.84^a^	24.88 ± 2.43	4.885	0.008
Final follow-up	36 months	25.16 ± 2.38	23.75 ± 3.05^a^	24.62 ± 2.54^b^	9.407	< 0.001

UBE-PLIF, unilateral biportal endoscopic posterior lumbar interbody fusion; PE-TLIF, percutaneous endoscopic transforaminal lumbar interbody fusion; OLIF, oblique lumbar interbody fusion; VAS, Visual Analog Scale; ODI, Oswestry Disability Index; JOA, Japanese Orthopaedic Association.

a PE-TLIF vs. UBE-PLIF, *P* < 0.05.

b OLIF vs. UBE-PLIF, *P* < 0.05.

**Figure 1:**
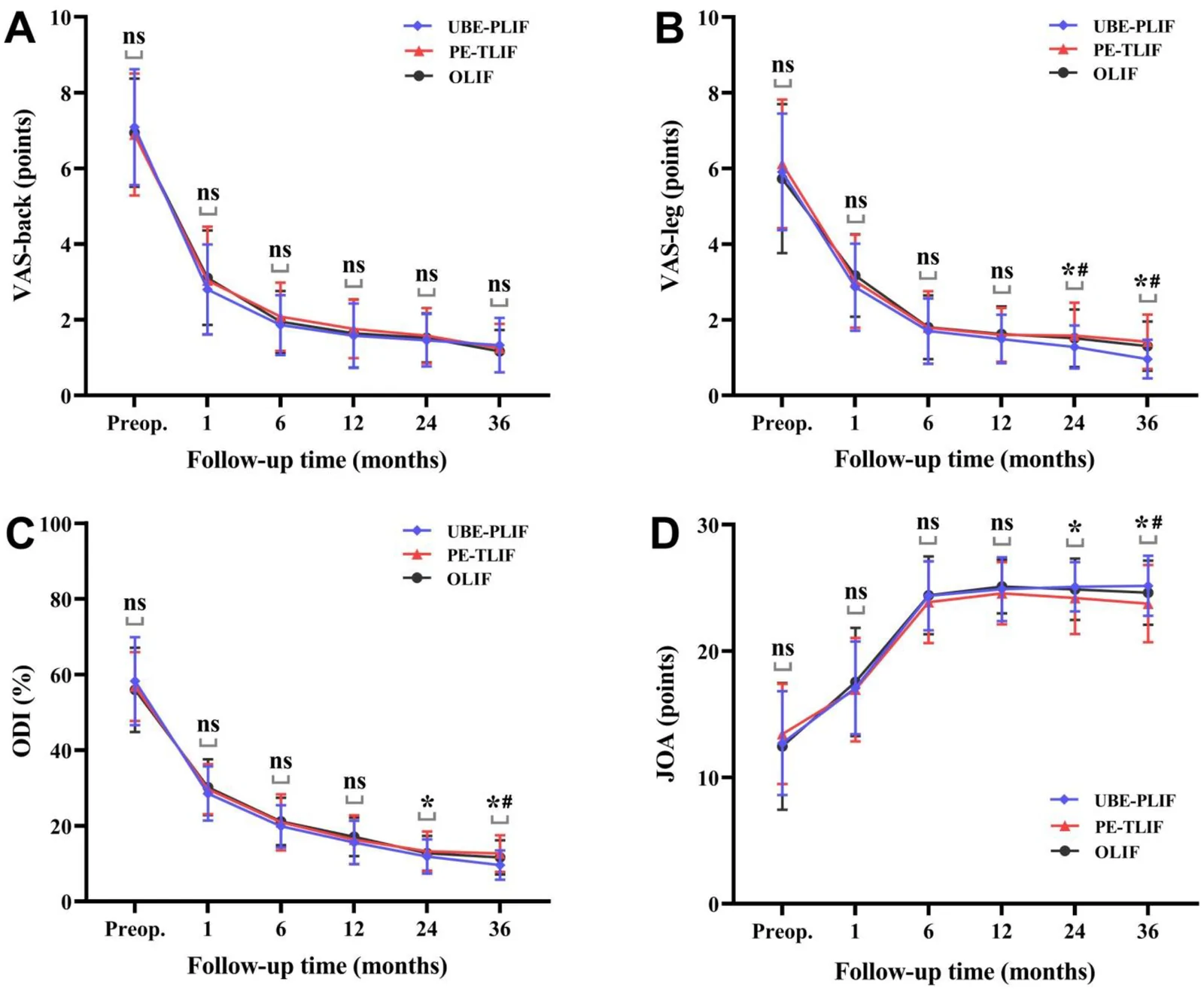
Comparisons of clinical outcomes across the three groups preoperatively (preop.) and at 1, 6, 12, 24, and 36 months postoperatively. **(A)** VAS-back scores; **(B)** VAS-leg scores; **(C)** ODI values; **(D)** JOA scores. ns, not statistically significant; * PE-TLIF vs. UBE-PLIF, *P* < 0.05; ^#^ OLIF vs. UBE-PLIF, *P* < 0.05. UBE-PLIF, unilateral biportal endoscopic posterior lumbar interbody fusion; PE-TLIF, percutaneous endoscopic transforaminal lumbar interbody fusion; OLIF, oblique lumbar interbody fusion; VAS, Visual Analog Scale; ODI, Oswestry Disability Index; JOA, Japanese Orthopaedic Association.

### Comparison of radiographic outcomes

No significant differences were observed among the three groups in preoperative intervertebral disc height (IDH), slip percentage, or lumbar lordosis angle (*P* > 0.05). Postoperatively, the OLIF group exhibited a significantly higher fusion rate than the PE-TLIF group at 6 months (*P* < 0.05). At 36 months, the OLIF group also showed greater IDH compared to the PE-TLIF group (*P* < 0.05). No other significant differences in radiographic parameters were detected at the remaining timepoints (*P* > 0.05), as shown in Table 4 and Figure 2.

**Table 4:** Comparison of radiographic outcomes among the three groups [($\bar{x}$ ± s), n(%)].

Radiographic outcomes		UBE-PLIF (*n* = 133)	PE-TLIF (*n* = 132)	OLIF (*n* = 137)	*F/χ^2^*	*P*
IDH (mm)						
Preoperatively	Admission	6.87 ± 2.03	7.21 ± 1.91	7.03 ± 2.12	0.937	0.393
Postoperatively	1 month	11.70 ± 1.53	11.93 ± 1.44	12.02 ± 1.61	1.564	0.211
	6 months	11.26 ± 1.59	11.38 ± 1.70	11.70 ± 2.03	2.196	0.113
	12 months	11.15 ± 1.68	11.30 ± 1.72	11.51 ± 1.90	1.410	0.245
	24 months	10.94 ± 1.74	10.85 ± 1.83	11.30 ± 2.01	2.205	0.112
Final follow-up	36 months	10.81 ± 1.56	10.74 ± 1.68	11.29 ± 1.75^a^	4.371	0.013
Slip percentage (%)						
Preoperatively	Admission	24.72 ± 6.83	23.61 ± 7.78	25.25 ± 8.14	1.620	0.199
Postoperatively	1 month	6.85 ± 3.21	7.38 ± 4.12	6.70 ± 3.36	1.328	0.266
	6 months	7.13 ± 3.60	7.72 ± 3.89	7.43 ± 3.75	0.821	0.441
	12 months	7.51 ± 3.38	8.38 ± 4.03	7.73 ± 3.92	1.890	0.152
	24 months	7.84 ± 4.12	8.75 ± 3.86	8.36 ± 4.11	1.699	0.184
Final follow-up	36 months	8.19 ± 2.98	9.04 ± 3.51	8.67 ± 4.03	1.923	0.147
Lumbar lordosis angle (°)						
Preoperatively	Admission	38.42 ± 13.36	41.49 ± 11.83	39.33 ± 13.15	2.010	0.135
Postoperatively	1 month	46.38 ± 6.91	45.92 ± 7.74	46.86 ± 9.27	0.459	0.632
	6 months	46.25 ± 7.38	45.58 ± 9.17	45.32 ± 10.50	0.372	0.690
	12 months	45.61 ± 8.93	43.82 ± 10.58	44.92 ± 8.08	1.264	0.284
	24 months	44.87 ± 10.12	45.90 ± 8.65	44.68 ± 11.03	0.586	0.563
Final follow-up	36 months	44.54 ± 8.70	43.61 ± 10.24	45.57 ± 8.82	1.504	0.223
Interbody fusion rate (%)						
Preoperatively	Admission	-	-	-	-	-
Postoperatively	1 month	-	-	-	-	-
	6 months	52 (39.10)	46 (34.85)	68 (49.64)^a^	6.459	0.040
	12 months	109 (81.95)	101 (76.52)	118 (86.13)	4.156	0.125
	24 months	127 (95.49)	122 (92.42)	132 (96.35)	2.297	0.317
Final follow-up	36 months	130 (97.74)	128 (96.97)	136 (99.27)	-^b^	0.372

UBE-PLIF, unilateral biportal endoscopic posterior lumbar interbody fusion; PE-TLIF, percutaneous endoscopic transforaminal lumbar interbody fusion; OLIF, oblique lumbar interbody fusion; IDH, intervertebral disc height.

a OLIF vs. PE-TLIF, *P* < 0.05; - Data not available.

b Fisher's exact test.

**Figure 2:**
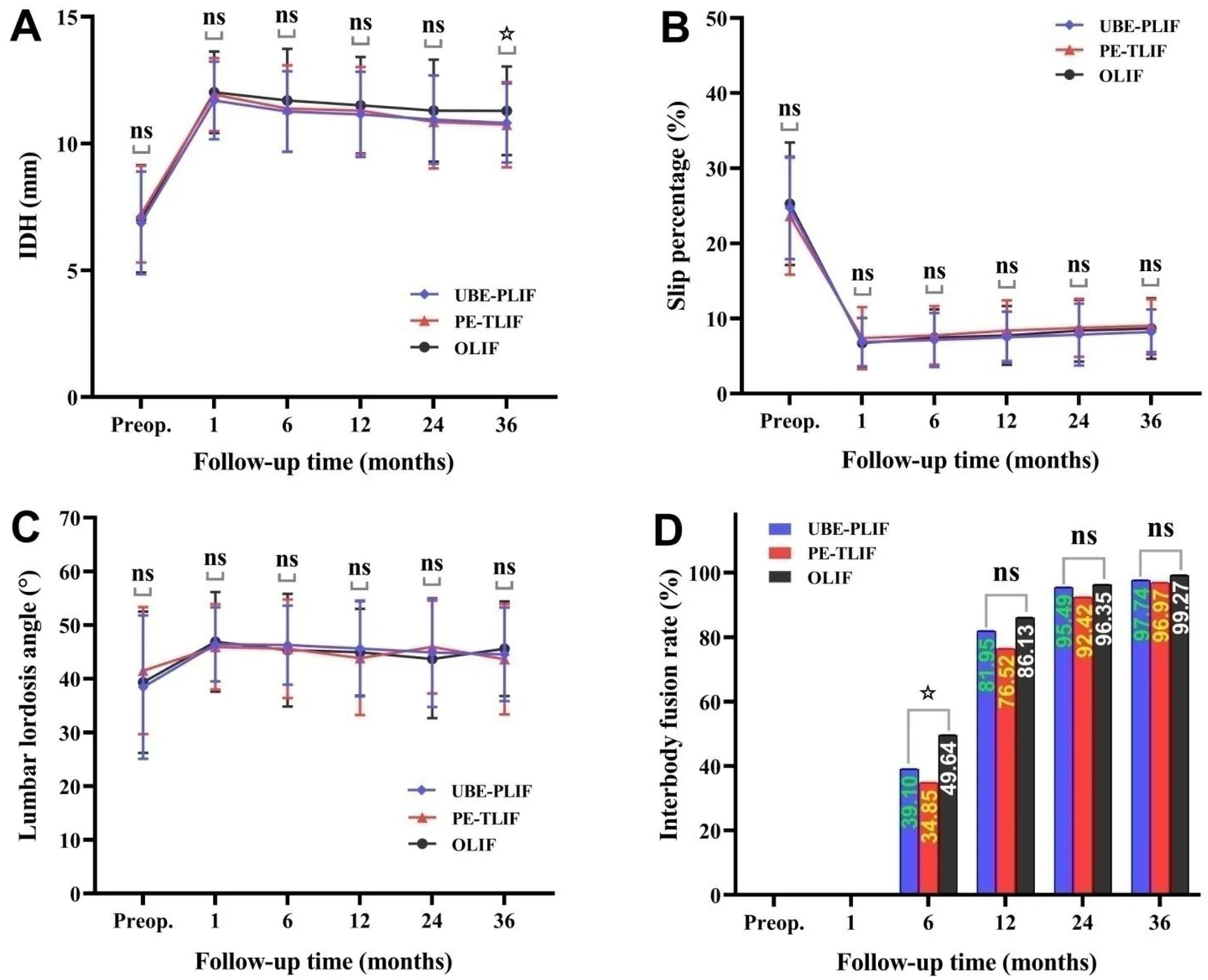
Comparisons of radiographic outcomes across the three groups preoperatively (preop.) and at 1, 6, 12, 24, and 36 months postoperatively. **(A)** IDH; **(B)** Slip percentage; **(C)** Lumbar lordosis angle; **(D)** Interbody fusion rate. ns, not statistically significant; ^⋆^ OLIF vs. PE-TLIF, *P* < 0.05. UBE-PLIF, unilateral biportal endoscopic posterior lumbar interbody fusion; PE-TLIF, percutaneous endoscopic transforaminal lumbar interbody fusion; OLIF, oblique lumbar interbody fusion; IDH, intervertebral disc height.

### Comparison of complications

The total rates of surgery-related complications were 13.53% (UBE-PLIF), 18.94% (PE-TLIF), and 16.79% (OLIF), comprising minor (10.53%, 15.15%, 13.87%) and major complications (3.01%, 3.79%, 2.92%), respectively. No significant intergroup differences were found for any complication category (*P* > 0.05), as shown in Table 5.

**Table 5:** Comparison of complications among the three groups [n(%)].

Complications and categories		UBE-PLIF (*n* = 133)	PE-TLIF (*n* = 132)	OLIF (*n* = 137)	*χ^2^*	*P*
Intraoperative	Minor complications					
	Small dural tear	2 (1.50)	5 (3.79)	1 (0.73)		
	Nerve root injury	3 (2.26)	2 (1.52)	2 (1.46)		
	Incomplete decompression	0 (0.00)	4 (3.03)	1 (0.73)		
	Psoas muscle injury	0 (0.00)	0 (0.00)	2 (1.46)		
	Subtotal	5 (3.76)	11 (8.33)	6 (4.38)	3.159	0.206
	Major complications					
	Large dural tear	1 (0.75)	2 (1.52)	1 (0.73)		
	Segmental vascular injury	0 (0.00)	0 (0.00)	2 (1.46)		
	Subtotal	1 (0.75)	2 (1.52)	3 (2.19)	-^a^	0.947
Postoperative	Minor complications					
	Transient palsy	4 (3.01)	2 (1.52)	5 (3.65)		
	Epidural hematoma	1 (0.75)	1 (0.76)	2 (1.46)		
	Superficial surgical site infection	0 (0.00)	2 (1.52)	1 (0.73)		
	Subtotal	5 (3.76)	5 (3.79)	8 (5.84)	0.901	0.637
	Major complications					
	Compressive epidural hematoma	1 (0.75)	1 (0.76)	0 (0.00)		
	Deep surgical site infection	0 (0.00)	1 (0.76)	0 (0.00)		
	Subtotal	1 (0.75)	2 (1.52)	0 (0.00)	-^a^	0.354
At follow-up	Minor complications					
	Screw loosening	2 (1.50)	1 (0.76)	0 (0.00)		
	Cage subsidence	1 (0.75)	2 (1.52)	1 (0.73)		
	Adjacent segment degeneration	0 (0.00)	1 (0.76)	2 (1.46)		
	Slip recurrence	1 (0.75)	0 (0.00)	2 (1.46)		
	Subtotal	4 (3.01)	4 (3.03)	5 (3.65)	0.115	0.944
	Major complications					
	Permanent palsy	1 (0.75)	1 (0.76)	0 (0.00)		
	Reoperation	1 (0.75)^b^	0 (0.00)	1 (0.73)^c^		
	Subtotal	2 (1.50)	1 (0.76)	1 (0.73)	-^a^	0.772
Total rate	Minor complications	14 (10.53)	20 (15.15)	19 (13.87)	1.323	0.516
	Major complications	4 (3.01)	5 (3.79)	4 (2.92)	0.194	0.907
	Total	18 (13.53)	25 (18.94)	23 (16.79)	1.431	0.489

UBE-PLIF, unilateral biportal endoscopic posterior lumbar interbody fusion; PE-TLIF, percutaneous endoscopic transforaminal lumbar interbody fusion; OLIF, oblique lumbar interbody fusion.

a Fisher's exact test.

b Reoperation for screw breakage.

c Reoperation for intervertebral osteolysis.

## Discussion

Minimally invasive lumbar interbody fusion has garnered global attention for treating LS and LSS. Among the various minimally invasive techniques, UBE-PLIF, PE-TLIF, and OLIF have attracted growing attention and are frequently employed at our institution. This study provides a comprehensive comparison of their surgical parameters, safety, and outcomes, revealing broadly comparable effectiveness alongside distinct characteristics in specific aspects. Our findings showed that the UBE-PLIF procedure was associated with a shorter total incision and reduced blood loss, whereas the OLIF approach was associated with longer incisions, greater blood loss, and higher drainage volumes. According to views from previous studies (24, 25), the better hemostatic performance in the UBE-PLIF group may be related to its higher irrigation pressure, which helps control minor bleeding, improves surgical field visualization, and enhances hemostatic efficiency. Furthermore, a systematic review demonstrated that UBE technique had a positive effect on reducing intraoperative blood loss and postoperative drainage volume, but this technique may prolong the operation time (26). However, we believe that operation time is largely dependent on the surgeon's learning curve and proficiency. As demonstrated by Shao et al. (27), UBE represents an effective minimally invasive spinal endoscopic technique for lumbar spinal diseases, offering reduced operation time after technique mastery. Moreover, studies on the learning curve of UBE have indicated that a minimum of 29 cases are required to achieve proficiency, while 41 cases are needed to reach a stable surgical success rate (28). Our surgical team's extensive experience, gained from performing hundreds of UBE procedures annually, has indeed led to significantly reduced operation times.

Regarding clinical outcomes, the superior leg pain relief and functional improvement (evidenced by VAS-leg, ODI, and JOA) in the UBE-PLIF group at later follow-ups might be partially explained by the enhanced visualization and maneuverability of the bilateral endoscopic approach, which could theoretically enable more thorough decompression (29–31). However, our study did not directly measure decompression adequacy (e.g., MRI cross-sectional area), so this interpretation remains speculative. In contrast, the lack of significant differences in VAS-back across all groups suggests that postoperative axial pain may be similarly influenced by the use of minimally invasive pedicle screw fixation and identical interbody fusion techniques. For radiographic outcomes, the overall comparability in IDH, slip percentage, lumbar lordosis angle, and fusion rate over 36 months might be attributable to three factors: all procedures were performed by an experienced surgical team; the restriction strategy based on age, BMI, and BMD may have reduced baseline heterogeneity; and most statistically significant differences fell below established Minimal Clinically Important Difference (MCID) thresholds (32, 33). Thus, we cautiously conclude that the three techniques are comparably effective in maintaining structural integrity and achieving fusion, a conclusion generally consistent with existing literature (34, 35). Unlike our study, those studies likely included some patients without LSS, resulting in better baseline functional scores (e.g., VAS, ODI, or JOA) than our results. A prior comparison of MIS-TLIF, Endo-TLIF, and OLIF reported that while all three techniques are effective for mild or moderate lumbar spondylolisthesis, OLIF had advantages including less blood loss, better IDH maintenance, and a higher early fusion rate (36). In our study, OLIF also showed a higher early fusion rate, but UBE-PLIF achieved modest advantages in blood loss and functional scores, suggesting its potential alternative role. Despite this difference, the postoperative recovery trajectories and final improvement magnitudes were similar during mid-term follow-up, indicating that all three techniques are feasible for LS and LSS.

The complication rate in lumbar interbody fusion surgery varies widely. A large-scale meta-analysis indicated that complication rates range from 4% to 54%, with a pooled overall incidence of 19% (37). Another study analyzing the American Board of Orthopedic Surgery database reported an overall complication rate of 22.2% among 6,841 fusion cases (38). In this study, complications were reported using a binary classification system, focusing on comparing surgery-related complications across the three groups. The overall complication rates were 13.53% (UBE-PLIF), 18.94% (PE-TLIF), and 16.79% (OLIF), which are generally consistent with these benchmark rates. The proportions of major complications within each group were 3.01% (UBE-PLIF), 3.79% (PE-TLIF), and 2.92% (OLIF), closely aligning with the 3.23% reported by Saleh et al. (39). Analysis of individual complications revealed that dural tears, transient palsy, nerve root injury, and epidural hematoma were the most common, a distribution similar to that described in other studies (40). However, cage subsidence, previously identified as a frequent cause of indirect decompression failure (41), occurred at lower rates in our cohort. One possible explanation may be that the use of pedicle screw fixation combined with sufficient bone grafting enhances segmental stability. While pedicle screw fixation inevitably increases surgical trauma and implant-related risks (e.g., malposition, loosening, or breakage), its use was justified in this study because enrolled patients had an unstable performance. For patients with stable LS without instability, decompression alone may be adequate in most cases, and treatment selection should consider other conditions such as slip grade, stenosis pattern, and patient-specific factors. Only two reoperations were required during three-year follow-up: one for screw breakage (UBE-PLIF) and one for intervertebral osteolysis (OLIF). Other complications, such as cage subsidence, adjacent segment degeneration, and slip recurrence, remained infrequent. Overall, these findings further supported the comparable safety profiles of the three techniques.

This study has several limitations. First, this retrospective analysis of a three-year prospective cohort shows that although surgical decisions followed recent guidelines, technique choice was influenced by patient anatomy, stenosis pattern, and surgeon preference, potentially introducing confounders and operator-related bias. To improve group comparability, a restriction strategy based on well-recognized confounders such as age, BMI, lumbar BMD, and surgeon experience (>100 cases) was applied. We acknowledge that more rigorous methods may minimize confounders and selection bias, including propensity score matching (PSM), inverse probability of treatment weighting (IPTW), stratified analysis, or multivariable regression for confounder adjustment, as well as mixed-effects models or generalized estimating equations (GEE) for analyzing repeated measurements. Employing these methods in future studies is needed to reduce the impact of confounders and bias. Second, although VAS-leg, ODI, and JOA reached statistical significance at 24 months, intergroup differences were small (approximately 0.5–2 points), below reported MCID thresholds (ODI: 10–12.5 points; VAS-leg: 2.0–2.8 points; JOA: 2.5 points) (33, 42, 43). Therefore, we conclude that all three techniques demonstrate comparable mid-term efficacy, with UBE-PLIF being non-inferior in pain relief and functional improvement. Prospective studies with extended follow-up and MCID-based analyses are needed to better interpret clinical relevance. Third, because most participants came from the same region and its surrounding areas, the geographically clustered sample may introduce regional selection bias, which should be considered when interpreting the results. Finally, the UBE-PLIF group was chosen as the reference because it is a newer technique with a smaller sample size, allowing greater matching flexibility. However, this approach may cause imbalance across groups, affect result reliability, and limit generalizability to real-world practice for the general population. Collectively, large-sample, multicenter randomized controlled trials with intention-to-treat analysis are required to confirm our findings.

## Conclusion

UBE-PLIF, PE-TLIF, and OLIF demonstrate comparable overall safety and efficacy in treating grade I-II LS with LSS. Compared with the other two techniques, UBE-PLIF is associated with less blood loss and modest advantages in selected late clinical outcomes. These findings suggest that UBE-PLIF is a viable alternative for this patient population. However, given the single-region retrospective design and methodological limitations, further validation through multicenter randomized controlled trials with longer follow-up is warranted.

## Data Availability

The raw data supporting the conclusions of this article will be made available by the authors, without undue reservation.

## References

[1] Alhosanie TN Klaib AF Al Hadidi FA Kanaan T Mohammad K Al-zurgan A. Prevalence of lumbar spondylolisthesis in adults and elderly patients with low back pain in the Jordanian community: radiographic findings. Eur Spine J. (2025) 34:2571–9. 10.1007/s00586-025-08963-1 40442514

[2] Yang H Cheng F Hai Y Liu Y Pan A. Unilateral biportal endoscopic lumbar interbody fusion enhanced the recovery of patients with the lumbar degenerative disease compared with the conventional posterior procedures: a systematic review and meta-analysis. Front Neurol. (2023) 13:1089981. 10.3389/fneur.2022.1089981 36703632 PMC9871470

[3] Lan Z Yan J Yang Y Xu Q Jin Q. A review of the main classifications of lumbar spondylolisthesis. World Neurosurg. (2023) 171:94–102. 10.1016/j.wneu.2022.12.104 36584893

[4] Ball JR Gallo MC Kebaish K Hang N Ton A Hernandez F. National trends in lumbar degenerative spondylolisthesis with stenosis treated with fusion versus decompression. Neurospine. (2024) 21:1068–77. 10.14245/ns.2448624.312 39765242 PMC11744538

[5] Tunç B Uzlu O Şahin ÖF Ünal E Yılmaz A Çağıl E. Sleep and quality of life before and after surgery in patients with low-grade lumbar spondylolisthesis. Med Sci Monit. (2026) 32:e950921. 10.12659/MSM.950921 PMC12811916 41486641

[6] Li Y Zheng S Wu Y Liu X Dang G Sun Y. Trends of surgical treatment for spinal degenerative disease in China: a cohort of 37,897 inpatients from 2003 to 2016. Clin Interv Aging. (2019) 14:361–6. 10.2147/CIA.S191449 30863029 PMC6388778

[7] Deshpande V Simpson E Caballero J Haddad C Smith J Gardner V. Cost-utility of lumbar interbody fusion surgery: a systematic review. Spine J. (2025) 25:1117–38. 10.1016/j.spinee.2024.12.027 39805471

[8] Ravindra VM Senglaub SS Rattani A Dewan MC Härtl R Bisson E. Degenerative lumbar spine disease: estimating global incidence and worldwide volume. Global Spine J. (2018) 8:784–94. 10.1177/2192568218770769 30560029 PMC6293435

[9] Kotil K Akçetin M Tari R Ton T Bilge T. Replacement of vertebral lamina (laminoplasty) in surgery for lumbar isthmic spondylolisthesis. A prospective clinical study. Turk Neurosurg. (2009) 19:113–20. 19431119

[10] Weber C Hermansen E Myklebust TÅ Banitalebi H Brisby H Brox JI. Comparison of patients operated for lumbar spinal stenosis with and without spondylolisthesis: a secondary analysis of the NORDSTEN trials. Spine (Phila Pa 1976). (2024) 49:1332–8. 10.1097/BRS.0000000000005038 38857371

[11] Pokorny G Amaral R Marcelino F Moriguchi R Barreira I Yozo M. Minimally invasive versus open surgery for degenerative lumbar pathologies: a systematic review and meta-analysis. Eur Spine J. (2022) 31:2502–26. 10.1007/s00586-022-07327-3 35871660 PMC9308956

[12] Sasaki M Kishima H. Standard techniques of spinal fusion for lumbar degenerative diseases. No Shinkei Geka. (2021) 49:1257–70. 10.11477/mf.1436204512 34879345

[13] Mohile NV Kuczmarski AS Lee D Warburton C Rakoczy K Butler AJ. Spondylolysis and isthmic spondylolisthesis: a guide to diagnosis and management. J Am Board Fam Med. (2022) 35:1204–16. 10.3122/jabfm.2022.220130R1 36526328

[14] Bussières A Cancelliere C Ammendolia C Comer CM Zoubi FA Châtillon C-E. Non-surgical interventions for lumbar spinal stenosis leading to neurogenic claudication: a clinical practice guideline. J Pain. (2021) 22:1015–39. 10.1016/j.jpain.2021.03.147 33857615

[15] Luo J Shen L Bao C Gao Z. Long-term comparative study of open-TLIF, MIS-TLIF, and UBE-TLIF in single-level degenerative lumbar spondylolisthesis. Eur J Med Res. (2026) 31:330. 10.1186/s40001-026-03897-w 41593715 PMC12918009

[16] Li X Liu J Liu Z. Comparison of the results of open PLIF versus UBE PLIF in lumbar spinal stenosis: postoperative adjacent segment instability is lesser in UBE. J Orthop Surg Res. (2023) 18:543. 10.1186/s13018-023-04038-3 37516831 PMC10386635

[17] Yang J Liu C Hai Y Yin P Zhou L Zhang Y. Percutaneous endoscopic transforaminal lumbar interbody fusion for the treatment of lumbar spinal stenosis: preliminary report of seven cases with 12-month follow-up. Biomed Res Int. (2019) 2019:3091459. 10.1155/2019/3091459 31019966 PMC6451828

[18] Sardhara J Singh S Mehrotra A Bhaisora KS Das KK Srivastava AK. Neuro-navigation assisted pre-psoas minimally invasive oblique lumbar interbody fusion (MI-OLIF): new roads and impediments. Neurol India. (2019) 67:803–12. 10.4103/0028-3886.263262 31347559

[19] Huang X Wang W Chen G Guan X Zhou Y Tang Y. Comparison of surgical invasiveness, hidden blood loss, and clinical outcome between unilateral biportal endoscopic and minimally invasive transforaminal lumbar interbody fusion for lumbar degenerative disease: a retrospective cohort study. BMC Musculoskelet Disord. (2023) 24:274. 10.1186/s12891-023-06374-1 37038129 PMC10088165

[20] Bao BX Zhou JW Yu PF Chi C Qiang H Yan H. Transforaminal endoscopic discectomy and foraminoplasty for treating central lumbar stenosis. Orthop Surg. (2019) 11:1093–100. 10.1111/os.12559 31714030 PMC6904652

[21] You X Zhao B Zhang T Wang Y Xu C Yuan J. Clinical efficacy of unilateral dual-channel endoscopic lumbar interbody fusion for lumbar spondylolisthesis with spinal scoliosis. Orthop Surg. (2024) 16:1134–42. 10.1111/os.14046 38520125 PMC11062877

[22] Ding JZ Kong C Sun XY Lu SB. Perioperative complications and risk factors in degenerative lumbar scoliosis surgery for patients older than 70 years of ge. Clin Interv Aging. (2019) 14:2195–203. 10.2147/CIA.S218204 31908430 PMC6924653

[23] De la Garza Ramos R Choi JH Naidu I Benton JA Echt M Yanamadala V. Racial disparities in perioperative morbidity following oncological spine surgery. Global Spine J. (2023) 13:1194–9. 10.1177/21925682211022290 34124959 PMC10416608

[24] Lee DH Lee D-G Park CK Jang J-W Hwang JS Kim JY. Saving stabilizing structure treatment with bilateral-contralateral decompression for spinal stenosis in degenerative spondylolisthesis using unilateral biportal endoscopy. Neurospine. (2023) 20:931–9. 10.14245/ns.2346504.252 37798987 PMC10562235

[25] Zhang Z Shao J Meng H Liu S Fan Z Lin J. Modifiable risk factors for perioperative hidden blood loss in unilateral biportal endoscopic surgery: a systematic review and meta-analysis. Wideochir Inne Tech Maloinwazyjne. (2025) 20:235–43. 10.20452/wiitm.2025.17986 41209752 PMC12590373

[26] Li Y Gao SJ Hu X Lin SS. Comparison of efficacy between unilateral biportal endoscopic lumbar fusion versus minimally invasive transforaminal lumbar fusion in the treatment of lumbar degenerative diseases: a systematic review and meta-analysis. Medicine (Baltimore). (2023) 102:e34705. 10.1097/MD.0000000000034705 37653732 PMC10470694

[27] Shao J Fan Z Meng H Fei Q. Learning curve and complications of unilateral biportal endoscopy-unilateral laminectomy bilateral decompression for lumbar spinal stenosis. Wideochir Inne Tech Maloinwazyjne. (2024) 19:489–97. 10.20452/wiitm.2024.17905 40123727 PMC11927538

[28] Guo W Ye J Li T Yu Y Fan X. Evaluation of the learning curve and complications in unilateral biportal endoscopic transforaminal lumbar interbody fusion: cumulative sum analysis and risk-adjusted cumulative sum analysis. J Orthop Surg Res. (2024) 19:194. 10.1186/s13018-024-04674-3 38509573 PMC10956305

[29] Chen Y Lin W Lei S You Y Zhang X Ma Y. Comparing the efficacy and safety of unilateral biportal endoscopic decompression with percutaneous endoscopic lumbar decompression for lumbar degenerative diseases: a meta-analysis. World Neurosurg. (2024) 187:e383–98. 10.1016/j.wneu.2024.04.093 38657790

[30] Zhang Z Cui W Dong YB Yu Y. Clinical efficacy of unilateral biportal endoscopy with unilateral laminotomy for bilateral decompression. World Neurosurg. (2025) 193:142–4. 10.1016/j.wneu.2024.10.066 39455007

[31] Meng H Su N Lin J Fei Q. Comparative efficacy of unilateral biportal endoscopy and micro-endoscopic discectomy in the treatment of degenerative lumbar spinal stenosis: a systematic review and meta-analysis. J Orthop Surg Res. (2023) 18:814. 10.1186/s13018-023-04322-2 37907922 PMC10619222

[32] Nie JW Hartman TJ MacGregor KR Oyetayo OO Zheng E Singh K. Establishing minimum clinically important difference for patient-reported outcome measures in patients undergoing lateral lumbar interbody fusion. Acta Neurochir (Wien). (2023) 165:325–34. 10.1007/s00701-022-05428-y 36602614

[33] Wang D Chen X Han D Wang W Kong C Lu S. Radiographic predictors of reaching minimal clinically important difference following lumbar fusion surgery in patients with degenerative lumbar spondylolisthesis. Eur Spine J. (2024) 33:1786–95. 10.1007/s00586-023-08051-2 38093000

[34] Gutierrez YV Martin B Nwose J Sepulveda AL Shaju RA Ahmad A. Exploring the efficacy of Various minimally invasive surgery (MIS) techniques in spinal fusion for degenerative spondylolisthesis: a systematic review. Cureus. (2025) 17:e88723. 10.7759/cureus.88723 40861602 PMC12375262

[35] Caelers IJMH Droeghaag R De Kunder SL Most J Rijkers K Bartels RHMA. Transforaminal versus posterior lumbar interbody fusion for symptomatic single-level spondylolisthesis (LIFT): a multicentre controlled, patient blinded, randomised non-inferiority trial. Lancet Reg Health Eur. (2024) 43:100964. 10.1016/j.lanepe.2024.100964 39022428 PMC11253221

[36] Song Z Zhang Z Zheng J Zhang K Wang F Ran M. Short-term and mid-term evaluation of three types of minimally invasive lumbar fusion surgery for treatment of L4/L5 degenerative spondylolisthesis. Sci Rep. (2024) 14:4320. 10.1038/s41598-024-54970-5 38383595 PMC10881486

[37] Ma H Zhang F Ying Q Pan B Li Y Ge H. Long-Term clinical and imaging results of oblique lateral interbody fusion for degenerative lumbar spondylolisthesis: a systematic review and meta-analysis. Orthop Surg. (2023) 15:400–12. 10.1111/os.13588 36479592 PMC9891977

[38] Childs S Bakhsh W Thirukumaran C Emery S Rubery P Mesfin A. Trends in lumbar interbody fusion: a study of American board of orthopedic surgery (ABOS) candidate data. Surg Neurol Int. (2023) 14:426. 10.25259/SNI_502_2023 38213447 PMC10783666

[39] Saleh A Thirukumaran C Mesfin A Molinari RW. Complications and readmission after lumbar spine surgery in elderly patients: an analysis of 2,320 patients. Spine J. (2017) 17:1106–12. 10.1016/j.spinee.2017.03.019 28385519

[40] Ju CI Lee SM. Complications and management of endoscopic spinal surgery. Neurospine. (2023) 20:56–77. 10.14245/ns.2346226.113 37016854 PMC10080410

[41] Ge T Ao J Li G Lang Z Sun Y. Additional lateral plate fixation has no effect to prevent cage subsidence in oblique lumbar interbody fusion. J Orthop Surg Res. (2021) 16:584. 10.1186/s13018-021-02725-7 34635143 PMC8507130

[42] Maki S Furuya T Katsumi K Nakajima H Honjoh K Watanabe S. Multimodal deep learning-based radiomics approach for predicting surgical outcomes in patients with cervical ossification of the posterior longitudinal ligament. Spine (Phila Pa 1976). (2024) 49:1561–9. 10.1097/BRS.0000000000005088 38975742

[43] Bayram Y Karataş ME. Radiographic and clinical outcomes at 12 months following full endoscopic interlaminar decompression for grade 1 degenerative lumbar spondylolisthesis. Sci Rep. (2025) 15:44565. 10.1038/s41598-025-28256-3 41444313 PMC12738734

